# Challenges in the Forging of Steel-Aluminum Bearing Bushings

**DOI:** 10.3390/ma14040803

**Published:** 2021-02-08

**Authors:** Bernd-Arno Behrens, Johanna Uhe, Tom Petersen, Christian Klose, Susanne E. Thürer, Julian Diefenbach, Anna Chugreeva

**Affiliations:** 1 Institut für Umformtechnik und Umformmaschinen (Forming Technology and Machines), Leibniz Universität Hannover, An der Universität 2, 30823 Garbsen, Germany; behrens@ifum.uni-hannover.de (B.-A.B.); uhe@ifum.uni-hannover.de (J.U.); petersen@ifum.uni-hannover.de (T.P.); diefenbach@ifum.uni-hannover.de (J.D.); 2 Institut für Werkstoffkunde (Materials Science), Leibniz Universität Hannover, An der Universität 2, 30823 Garbsen, Germany; klose@iw.uni-hannover.de (C.K.); thuerer@iw.uni-hannover.de (S.E.T.)

**Keywords:** tailored forming, hybrid components, bimetal bearing bushing, induction heating, intermetallic phases, compound forging

## Abstract

The current study introduces a method for manufacturing steel–aluminum bearing bushings by compound forging. To study the process, cylindrical bimetal workpieces consisting of steel AISI 4820 (1.7147, 20MnCr5) in the internal diameter and aluminum 6082 (3.2315, AlSi1MgMn) in the external diameter were used. The forming of compounds consisting of dissimilar materials is challenging due to their different thermophysical and mechanical properties. The specific heating concept discussed in this article was developed in order to achieve sufficient formability for both materials simultaneously. By means of tailored heating, the bimetal workpieces were successfully formed to a bearing bushing geometry using two different strategies with different heating durations. A metallurgical bond without any forging defects, e.g., gaps and cracks, was observed in areas of high deformation. The steel–aluminum interface was subsequently examined by optical microscopy, scanning electron microscopy (SEM) and energy dispersive spectroscopy (EDS). It was found that the examined forming process, which utilized steel–aluminum workpieces having no metallurgical bond prior to forming, led to the formation of insular intermetallic phases along the joining zone with a maximum thickness of approximately 5–7 µm. The results of the EDS analysis indicated a prevailing Fe_x_Al_y_ phase in the resulting intermetallic layer.

## 1. Introduction

Bulk metal forming is the key technology for manufacturing technical components with complex geometries, continuous grain flow and outstanding mechanical properties [1]. On an industrial scale, workpieces made of a single material are commonly used for conventional forging processes. However, monomaterial products might not be able to meet conflicting requirements such as the ever-increasing demand for lightweight construction, high strength, extended functionality and resource efficiency while maintaining low costs. Motivated by new economic and technical trends, manufacturers are steadily becoming interested in developing new technological solutions to reduce resource and energy consumption. In this context, multimaterial design is an effective approach [2]. When combining lightweight and high-performance materials in a single component, it is possible to lower the total weight and produce multimaterial components showing improved wear characteristics on functional surfaces at the same time.

The current study shows the potential for creating hybrid bearing bushings made of steel and aluminum and deals with the key challenges accompanying the forming stage. The forming process examined in this work was achieved via compound forging, where forming and joining are combined within a single step. As shown below in the literature survey, the already existing works on compound forging of steel–aluminum joints were primarily focused on upsetting of simplified geometries, e.g., coaxially arranged raw parts, where aluminum was often used as a core material. Within the current study, an automated forging process for a complex geometry of a bearing bushing with a high deformation ratio was realized. The main challenge of the forming process was to achieve a high radial expansion in the upper part, where extremely high formability is required because of tensile stress state. Due to the specific application requirements, it was necessary to place the steel on the inside of the component. As a result, an induction heating strategy with an inner coil had to be developed and was successfully implemented into the forging process. An automated process setup in a forging cell was used in order to achieve a high reproducibility as well as to minimize the temperature losses during the workpiece transfer, which are crucial in case of the investigated material combination. Due to the dissimilar forming behavior of steel and aluminum, specific heating strategies were necessary in order to achieve the required temperature gradient with the typical forming temperatures for both materials. Therefore, one may conclude that the main issue in forging dissimilar materials lies in the heating of bimetal workpieces. The objective of this investigation was to define the process window, which allows forming of hybrid bearing bushings. The required temperature gradients were achieved by means of induction heating. The developed heating strategies were subsequently used for the forging tests. The main objective of the forming process was to achieve a sufficient bond quality within a compound without separations, cracks or other material defects. At the end of this study, a comprehensive characterization of the bonding zone is carried out by means of SEM, and EDS.

The current work demonstrates supplementary examinations in the framework of the Collaborative Research Center 1153 (CRC 1153) “Tailored Forming”, which focuses on the forming of previously joined materials. In future examinations, the results presented in this paper will be compared to the forging of coextruded steel–aluminum tubes where a metallurgical bond between the joining partners already exists.

## 2. Survey of Current Literature

The forging of hybrid components can be performed with two types of processes, which differ in the initial state of the workpieces. The first one includes the forming of previously joined workpieces aimed at changing the shape of the workpiece and the thermomechanical treatment of the joining zone. This topic is the focus of the previously mentioned CRC 1153 [3]. The second one combines both joining and forming operations in a single step and is called compound forging. This method is examined in this paper as a preliminary study for the tailored forming of hybrid bearing bushings in order to show the influence of the forming on the joint without a prior joining operation.

The main challenge in compound forging is the production of a satisfactory joint between dissimilar materials, such as steel and aluminum. Diverse material and process related factors (e.g., flow stresses, plastic strain, acting forming forces, temperatures, surface properties and thermal expansion) influence the joining zone’s resulting quality. Moreover, a joint flow of materials also depends on the geometry of the combined raw parts (e.g., their arrangements, diameter ratios between shell and core) as well as the geometry of forging dies [4]. Kong et al. investigated the production of steel–aluminum compounds (AISI 316L/6063) by forge welding using different process parameters such as temperature, part geometry and forging speed [5]. Among them, the forming temperature had the greatest influence on the final joint quality and bond strength. The work of Wohletz and Groche deals with the joining of steel–aluminum combinations (AISI 1020/6082) by plastic deformation combining forward and cup extrusion at ambient and elevated temperatures [6]. The obtained joints demonstrated enhanced bond quality with increasing forming temperatures. At the same time, however, the increasing formation of oxide scale at elevated temperatures had a negative effect on the joining zone. Politis et al. studied the flow behavior of bimetal gears made of BS 970 230M07 steel jacket and 6082 aluminum core by numerical simulation and experiments [7]. It was established that interfacial and tooling friction provides an axial lock. Using different thicknesses of the steel, it was found that the formation of thin rings leads to an excessive material thinning, which results in local separations allowing the core material to come in contact with the die. Wu et al. carried out a similar research using a material combination of steel AISI 8620 and aluminum alloy 2014 [8]. Various gap sizes and height differences between core and jacket were investigated in order to define a processing window. Estrin et al. [9] presented an interesting approach for bimetal components with the focus on complex structures of reinforcing elements, which can be implemented by means of several plastic deformations. The research paper in question demonstrates an example based on an experiment that entails a spiral-layered structure consisting of aluminum encased in a copper cylinder. To achieve this structure, the process of forging with rotating forging die was used. Chavdar et al., as well as Goldstein et al. studied the hot forging and hot hydroforging of aluminum workpieces completely encapsulated within a steel shell using tailored heating strategies [10,11]. The forming was performed at conventional forging temperatures for steel, while the aluminum core was formed in a semisolid or molten state.

**Figure 1:**
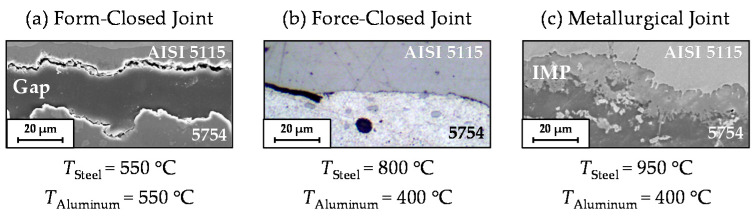
Classification of the resulting bond type depending on process temperatures during compound forging of AISI 5115 steel and aluminum 5754 [13]: (**a**) form-closed joint; (**b**) force-closed joint; (**c**) metallurgical joint.

Depending on the properties and contact conditions in the interface zone, there are three different types of joints that can be identified after compound forging. Figure 1 illustrates the classification of the bonding quality according to the results of previous basic research performed by the authors [12]. Here, the aluminum core (5754) and steel jacket (AISI 4820), which were assembled with a tight fit, were joined together by upsetting at different forming temperatures. The corresponding temperatures were measured at reference points in the centers of both the steel and the aluminum parts. At similar temperatures, aluminum has a higher thermal expansion and consequently, a higher shrinkage during cooling compared with steel. Accordingly, the forming of the examined steel–aluminum workpieces with homogeneous temperature distribution led to a separation of the materials in particular areas of the bimetal component (Figure 1a). The formation of a shrinking gap between the two materials was compensated for by an inhomogeneous heating strategy. With a steel temperature of 800 °C and an aluminum temperature of 400 °C, the gap between the materials was eliminated after cooling, and a force-closed joint between the materials was observed (Figure 1b). With a further increase in steel temperature to 950 °C, an intermetallic phase (IMP) was detected, achieving a metallurgical joint between steel and aluminum (Figure 1c).

The phase diagram of the binary system Al–Fe indicates six intermetallic phases [14]. Their main characteristics have been mentioned in various studies and are summarized in Table 1. All intermetallic phases have complex lattice structures that prevent easy dislocation slip, which leads to high hardness values (Table 1). Therefore, the thickness of the intermetallic layer is the key factor for the local and global quality of the component. Due to the low strength and high brittleness of intermetallic phases, the formation of thick intermetallic layers can lead to crack initiation in the joining zone and adversely affect the mechanical properties. As proved in previous research, the thickness of the intermetallic layers in Al–Fe compounds should not exceed 10 µm [15,16].

## 3. Materials and Methods

### 3.1. Initial and Final Geometry

**Figure 2:**
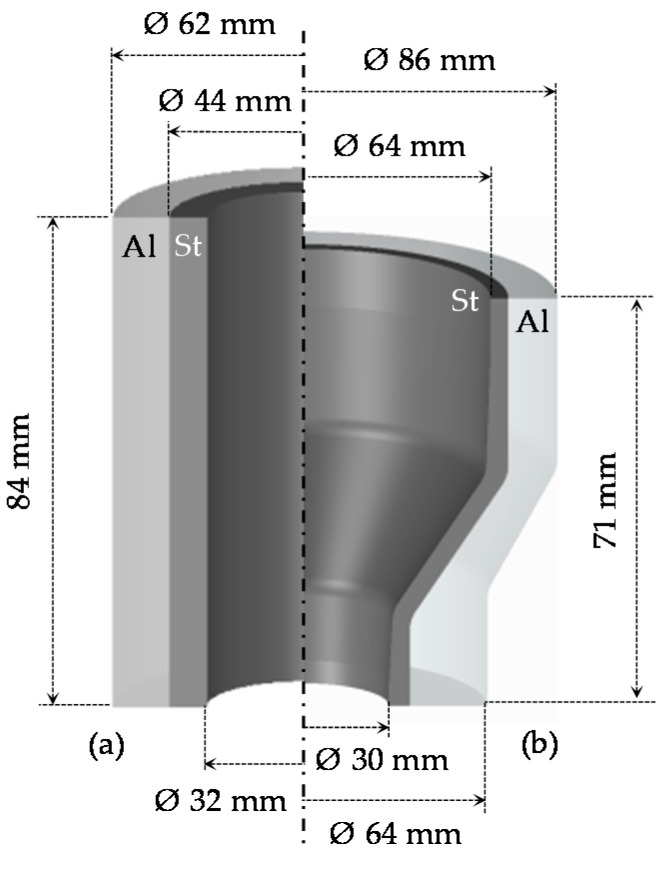
Examined geometries of a hybrid workpiece (**a**) and final bearing bushing (**b**) consisting of steel (St) and aluminum (Al).

In this work, bimetal workpieces including two coaxially arranged tubular components were used to produce bearing bushings (Figure 2). The steel tube (AISI 4820) was arranged on the inside to provide a wear-resistant surface for the high rolling stresses resulting from the contact with the bearing balls. The aluminum alloy 6082 was used in the peripheral area where the stresses are lower, in order to reduce total part weight. The workpieces were fabricated by machining, fitted together with a tolerance H7/g6 for a diameter of 44 mm (clearance fit) without metallurgical bonding, and subsequently formed to the final geometry. The steel thickness investigated in this work was chosen by analogy with the coextrusion process, which produces hybrid workpieces with initial bonds for further investigation, in order to be able to make a comparison [20].

### 3.2. Heating Strategy

For a flawless forming process, the steel and aluminum components must be heated to their material-specific forging temperatures in order to achieve sufficient formability. For this reason, an inhomogeneous temperature distribution within bimetal workpieces is required [21]. To avoid forming defects (e.g., cracks) and the shrinkage described above, the temperature of the steel part should be in the warm or hot forming temperature range. However, the maximum possible temperature for steel is limited due to aluminum’s onset-temperature (solidus temperature) of approximately 580 °C. Reaching different temperatures within a bimetal workpiece represents the main issue in the forging of dissimilar materials. The required temperature gradients can be achieved by induction heating, where the heat energy is generated by eddy currents. With an appropriate design of induction coil and relevant process parameters (i.e., power, frequency and heating time), the process can be tailored to the individual geometry of the bimetal workpieces. Due to the skin-effect occurring at middle and high operating frequencies, the highest current density is primarily concentrated on the workpiece surface which is subject to the highest thermal input. In the context of compound forging, this phenomenon can be used to generate heat in hybrid semifinished products. While the steel part is heated inductively, the aluminum part has its forming temperature from the heat dissipated by the steel. In order to achieve the required maximum gradient despite the high thermal conductivity of the aluminum, the heat transfer between the two materials has to be limited, which in this case should be achieved by selecting an appropriate clearance fit (see Section 3.1).

**Figure 3:**
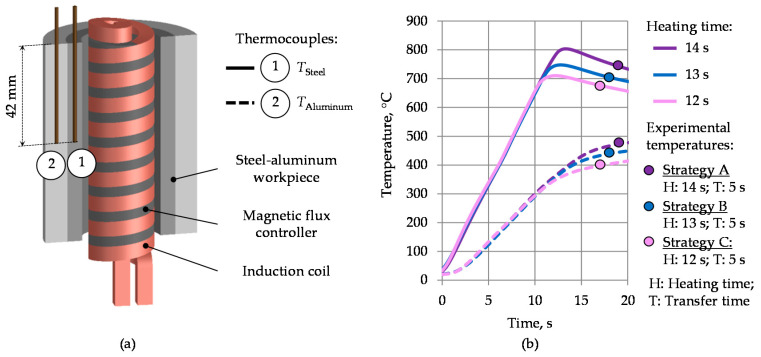
Schematic illustration of the heating concept with an internal induction coil (**a**) and corresponding temperature curves for varying heating time (**b**).

For the heating experiments, a middle-frequency generator Trumpf TruHeat MF 3040 (TRUMPF GmbH + Co. KG, Ditzingen, Germany) with a maximum power of 40 kW was used. The heating process was controlled by means of value specifications via a percentage of the maximum voltage. During the heating tests, time–temperature curves were recorded for the reference points in steel and aluminum. The measurement directly at the interface zone delivered implausible results, presumably due to rough local resolution of the used thermocouples. In order to be able to estimate prevailing temperature gradients, two thermocouples were placed as shown in Figure 3a. For the measurements, mineral-insulated thermocouples (type K, NiCr-Ni) with a stainless-steel sheath (Ø 1.5 mm) were used.

According to the workpiece geometry (Figure 2a), an induction heating concept with an internal induction coil was designed (Figure 3a). Compared to external induction heating, this concept results in extremely low efficiencies at high power demands [22]. For this reason, the inductor was improved by placing a soft magnetic flux controller Alphaform^®^ (Fluxtrol Inc., Auburn Hills, MI, USA) around the turns inside the coil. As a consequence of this improvement, the induced eddy currents are concentrated on the steel surface, reducing the additional power losses. This resulted in a significant increase in heating efficiency and a reduction in heating time. To achieve the highest possible temperature difference between steel and aluminum in a short time, the heating tests were carried out at a power level of 100%. The temperature curves obtained with the heating concept described above are shown in Figure 3b. Three heating strategies with various heating times of 12, 13 and 14 s resulted in different aluminum and steel temperatures and were used for the subsequent forging tests. The maximum possible time of the heating process was limited to 14 s. A longer heating duration would lead to the melting of aluminum due to exceeding the solidus temperature at approximately 580 °C after the equalizing of steel and aluminum temperatures [23]. The forging temperatures, including the transfer time of about 5 s after the intensive heating phase, are marked on the graph for each heating strategy—A, B and C with purple, blue or pink dots, respectively.

While developing and selecting heating strategies appropriate for the forming process, special attention was paid to the specific thermal expansion of the materials contained in the bimetal workpieces. After the cooling of the forgings, the jacket material has to be shrunk onto the internal component in order to avoid a shrinking gap in the joining zone. At the same temperature, the thermal expansion coefficient of aluminum is 1.5–2 times higher than that of steel. With an inhomogeneous temperature distribution, the entire thermal expansion of the material can be adapted to the individual requirements depending on the workpiece geometry.

**Figure 4:**
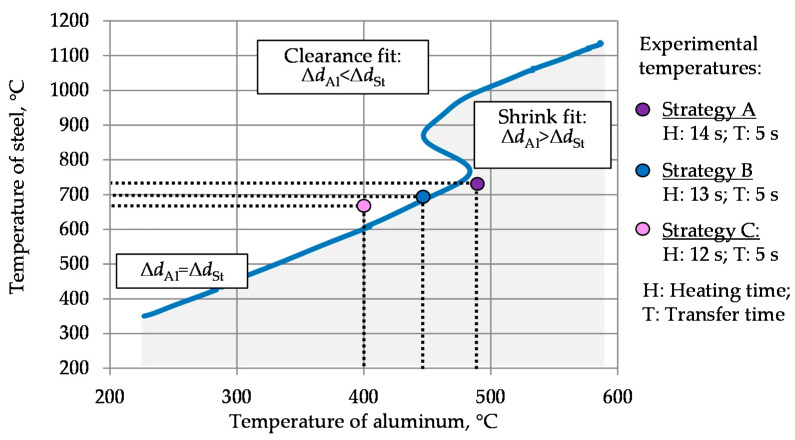
Process window regarding the thermal expansion of steel AISI 4820 and aluminum 6082.

The process window with regard to the thermal expansion of steel AISI 4820 (Δd_St_) and aluminum 6082 (Δd_Al_) can be illustrated with a curve represented in Figure 4. Based on the experimental dilatation data values measured at IFUM (Institut für Umformtechnik und Umformmaschinen), corresponding steel and aluminum temperatures with the equal thermal expansion were defined for each temperature. Here, the blue line corresponds to the temperature combinations at which steel and aluminum would exhibit equal thermal expansion and thus would shrink together after cooling. Taking into account the geometry of the workpiece, it is required that the shrinkage of aluminum after forming and cooling (Δd_Al_) is higher than that of steel (Δd_St_). When cooling from temperatures above this curve (e.g. 800 °C and 400 °C for steel and aluminum, respectively), the steel core will shrink more than the aluminum shell and could delaminate from the aluminum due to its more intensive thermal shrinking. For temperature combinations under this curve (e.g. 800 °C and 500 °C for steel and aluminum, respectively), the aluminum shell will shrink more and press the steel core from outside or even break due to arising thermal expansion stresses. To achieve this state, the temperatures of steel and aluminum should be below the blue line (grey area). Hence, the temperatures obtained by strategy A comply with the process window defined above. With strategy B, the forming temperatures are on the blue line, which relates to similar thermal expansion for both steel and aluminum. Strategy C resulted in a forming temperature beyond the blue line, failing to meet the criteria set by the process window.

### 3.3. Forging Process

**Figure 5:**
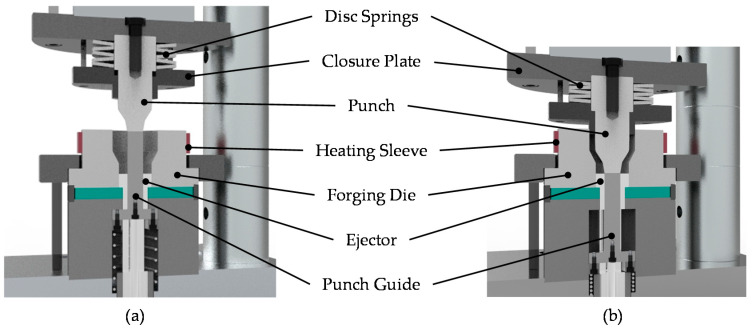
Forging tool system for forming of bearing bushings: beginning of the process (**a**); and end of the process (**b**).

In the present study, the forging tests were carried out on a screw press Lasco SPR 500 (Lasco Umformtechnik GmbH, Coburg, Germany) with a maximum capacity of 40 kJ. The forming process is performed in a single stage by closed-die forging. The material flow and stress analysis of the forging dies were examined by FEM (finite element method) analysis in a previous publication [24]. The corresponding tool system is depicted in Figure 5. The outer and inner geometry of the bearing bushing are formed, respectively, by forging die and punch. The forging die was preheated to a temperature of 250 °C. The closure plate in the upper tool ensures the final component height. After forming, the forged bushing is automatically detached from the upper punch by means of the disk spring force and removed from the forging die by an ejector. Before the forming process, a lubricant Berulit 913 (Carl Bechem GmbH, Hagen, Germany) diluted with water in a ratio of 1:3 was applied to the semifinished products. For this purpose, they were previously heated to a temperature of about 150 °C in a furnace and then coated with the mixture. After the water had evaporated, a thin layer of graphite and MoS_2_ remained on the surface. Subsequently, the coated workpieces were heated up to forging temperature by induction heating. A robot arm was utilized to handle and transport the workpieces to the forging press. As a result, reproducible process conditions for all forging tests can be achieved.

## 4. Results and Discussion

**Figure 6:**
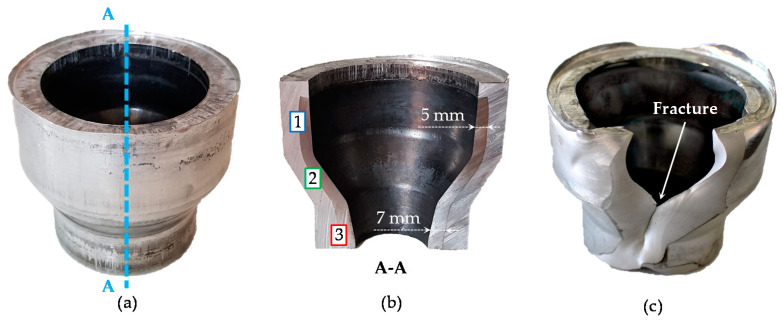
Bearing bushing forged with strategy A (**a**); its material distribution in longitudinal cross-section (**b**); material failure with the heating strategy C (**c**).

The heating strategies A, B and C defined above were used for the subsequent forging tests. A successful forming of the bearing bushings without any macroscopic defects was achieved by means of the heating strategies A and B. The final shape of the bearing bushing as well as material distribution in longitudinal cross-section achieved through strategy A are exemplarily illustrated in Figure 6a,b. The cross-section A–A is marked with a blue dash-line in Figure 6a. At forging temperatures for heating strategies A and B, the flow stress of aluminum is 5–7 times lower compared to that of steel as shown in Table 2. Due to this, aluminum has less flow resistance and flows over the steel in the upper part of the bearing bushings. It forms an undercut, which additionally withstands the axial movement between steel and aluminum components. The steel in the upper part undergoes a high radial expansion leading to a reduction of the wall thickness from 6 mm to 5 mm. In contrast to this, the wall thickness at the bottom of the steel part is increased to up to 7 mm, possibly by axial upsetting.

In contrast to strategy A and B, a material failure occurred during the forging with the heating strategy C (Figure 6c). The fractured surface demonstrated a blue discoloration on the steel side, which relates to the blue-brittleness effect caused by dynamic strain aging at insufficient low forming temperatures (for low carbon steels below the temperature of about 450 °C) [25]. Despite the fact that the temperature in the middle of the steel tube was in the typical warm forging range (about 670 °C) it can be hypothesized that the steel’s temperature close to the interface zone was lowered by heat exchange with the aluminum. Nearly similar temperatures in the interface zone might have a negative impact on the forming behavior of steel, if the aluminum temperature is below the minimal forging temperature of steel. As shown in Figure 3, the heating strategy C demonstrates the lowest aluminum forming temperature of about 400 °C compared to the other heating strategies and could be the reason for the material failure. In the strategies A and B, the forming temperatures of aluminum are above the critical temperature for steel of 450 °C. Compared to the strategy C, a higher formability was observed here, as expected.

**Figure 7:**
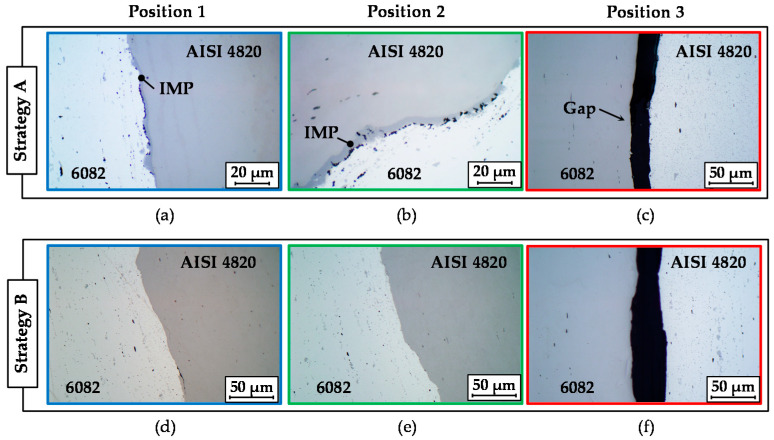
Micrographs of the joining zone between steel and aluminum in cross-section for the heating strategy A (**a**–**c**) and B (**d**–**f**) at the large diameter (**a**,**d**), in the inclination area (**b**,**e**) and at the small diameter (**c**,**f**).

For bearing bushings forged with the strategies A and B, the interface regions between the steel and the aluminum were examined using metallographic analysis at positions 1, 2 and 3 marked in Figure 6b. For both strategies, a complete bond without gaps, cracks and other defects was observed in the upper area with the largest diameter and in the inclination area, where high plastic deformation took place (Figure 7a,b,d,e), which can be associated with the local enlargement of the exterior surface area. For example, the change in diameter in the upper part from 44 mm to 73 mm leads to a circumferential elongation of approximately 66%. A high surface enlargement causes cracks and fractures of brittle passivation layers (oxide layers) due to normal and shear stresses in the interface zone [26]. Consequently, oxide-free material fills the emerging gaps and interacts with the opposite contact partner. High contact pressure due to plastic forming in combination with elevated temperatures intensifies local diffusion and ensures a reliable metallurgical bond between both materials [27]. Moreover, forging with heating strategy A results in the formation of intermetallic phases in the upper part of the bearing bushing as well as in the inclination area. This can be explained by aluminum’s higher temperature (about 490 °C) compared to strategy B (about 450 °C). In contrast, a low strain and a nearly absent contact surface enlargement in the bottom part of the bearing bushing counteract the bond formation. Consequently, the interface zone on the small diameter of the bearing bushing demonstrates a form-closed joint with some partial separations with a maximal gap size of approximately 30 μm for strategy A and 40 μm for strategy B (Figure 7c,f).

**Figure 8:**
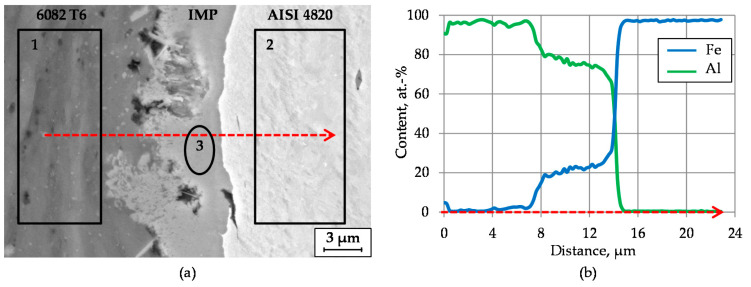
SEM micrograph (BSE mode) of the steel–aluminum joint achieved with heating strategy A with highlighted measurement regions (**a**) and EDS line scans with Fe and Al distributions (**b**).

The intermetallic phases formed in the upper part of the bearing bushing as well as in the inclination area have already been well distinguished by light microscopy due to their different colors compared to the base materials (Figure 8a,b). It can be stated that a metallurgical joint with an intermetallic compound layer has been formed at the steel–aluminum joining zone during the forging process. The intermetallic phases obtained were concentrated in separate insular areas and feature a nonuniform thickness along the joining zone [28]. The maximum thickness of the intermetallic phases varies between 4 µm and 7 µm. To evaluate the mechanical properties of interfacial components, a nanoindentation test was performed. The average nanohardness values of aluminum, steel and the intermetallic layer are 1.14, 5.08 and 11.1 GPa, respectively.

In order to characterize the steel–aluminum joint in detail, specimens extracted from the forged bearing bushings were investigated by means of scanning electron microscopy (SEM) using back-scattered electron (BSE) mode (Figure 8a). The dark regions next to the intermetallic layer in the middle of the picture include microscale pores, which probably occurred due to the melting of aluminum in the joining zone. The high heat input caused by plastic energy dissipation, the relative movement between steel and aluminum during forging as well as by the heat transfer from the steel are possible explanations for this phenomenon. The interaction of both materials initiated by friction effects and material flow in the joining zone is responsible for the formation of the intermetallic phases during the forming process.

The local compositions across the intermetallic compound layer between steel and aluminum were determined using energy dispersive X-ray spectroscopy (EDS). Figure 8b shows the material distribution of Fe and Al in atom percentage measured in line-scanning mode along the red arrow depicted in Figure 8a. It shows a mixture of both elements in the middle of the measured area and, as a result, is an indication for the presence of an intermetallic phase between steel and aluminum. The concentration of aluminum in the intermetallic layer slightly decreases in close proximity to the base material while the concentration of iron slightly increases. The average composition of the main components of the steel–aluminum joint was examined by local EDS analysis. The corresponding positions of the measurement regions (1, 2 and 3) are marked in Figure 8a. The contents of Al and Fe and other detected elements are listed in Table 3. The elemental distribution of the intermetallic phase is rich in both Fe and Al, and represents a mixture of the base materials.

High-aluminum intermetallic phases such as Fe_2_Al_5_ and FeAl_3_ are typical for joining processes with a short-time heat effect (e.g., compound forging) [29]. The formation of these phases starts above the recrystallization temperature of aluminum at approximately 350 °C to 400 °C. In this context, FeAl_3_ phase occurs in the temperature range between 350 °C and 500 °C [30]. The formation of a Fe_2_Al_5_ phase takes place at temperatures above 500 °C, whereas it can be partially transformed to FeAl_3_ during cooling [28].

**Figure 9:**
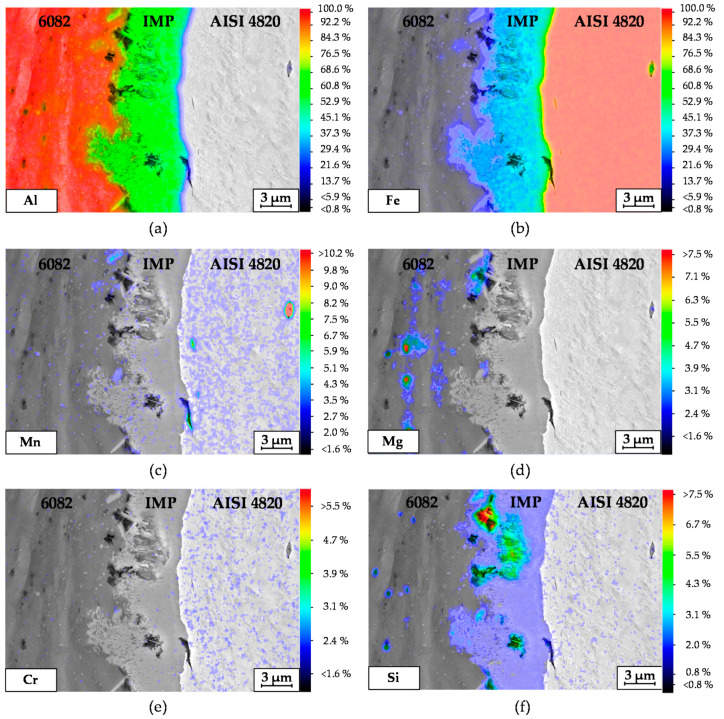
Quantitative EDS element-mapping images: Al (**a**); Fe (**b**); Mn (**c**); Mg (**d**); Cr (**e**) and Si (**f**) in wt.%.

The results of the EDS measurement (Figure 9) indicate an atomic percentage of 71.06 at.% of Al and 26.90 at.% of Fe. Regarding the Al to Fe ratio of about 5:2, the prevailing of Fe_2_Al_5_ intermetallic phase within the investigated forgings can be suggested. Additionally, the findings of hardness indentations with the measured average nanohardness of 11.1 GPa confirm this assumption. As considered by Matysik et al. by means of nanoindentation tests, the higher hardness values above 10 GPa correspond to the Fe_2_Al_5_ phase [31].

In addition, an EDS element-mapping analysis was carried out across the steel–aluminum joint shown in Figure 8a. Figure 9 depicts the corresponding results regarding the quantitative element distribution in weight percentage. Figure 8a,b shows Fe and Al distribution in a two-dimensional view. The maximum content of these elements can be seen in the base materials. The highlighted dots in the EDS mapping of Figure 9c,d correspond to the elements Mn and Mg, respectively. It is clearly visible that Mg and Mn inclusions are present in the aluminum base material. A red spot detected in Figure 9c corresponds to a MnS inclusion in steel. The higher concentration of dots on the right side in Figure 9c,e reflects the higher content of Mn and Cr in steel compared to aluminum. These observations correspond to the results shown in Table 3.

## 5. Conclusions

The manufacturing of bimaterial bearing bushings made of steel and aluminum by compound forging was investigated in the current study. Specific conclusions from this work include:

- The design of an appropriate heating strategy represents a key issue and one of the main challenges for the forming of dissimilar material combinations. Therefore, the bimetal workpieces have to be heated inhomogeneously in order to ensure specific forging temperatures for both of materials;
- Within this work, a specific heating concept in accordance with geometry and material combination was developed and realized by means of induction heating. With the temperature gradients achieved with heating strategies A and B, it was possible to ensure a sufficient formability for steel–aluminum workpieces assembled with a clearance fit. Subsequently, inhomogeneously heated hybrid workpieces were successfully formed to bearing bushings. Following strategy C, forming at inappropriate temperatures results in material fracture;
- With the strategies A and B, a complete metallurgical joint was observed in the upper part of the forgings exposed to high deformation. Moreover, insular intermetallic phases along the joining zone were observed with strategy A. By contrast, insufficient bonding with some partial separations with a maximal gap size of 40 μm was indicated in the bottom part with a lower deformation;
- A metallographical study including SEM and EDS investigations revealed an intermetallic phase of Fe_x_Al_y_ type with a thickness of approximately 4–7 µm in the steel–aluminum joint after the forming process with strategy A;
- According to the determined atomic percentage of 71.06 at.% for aluminum and 26.90 at.% for iron, the first assumptions indicate the prevalence of Fe_2_Al_5_ within the investigated intermetallic layer. This assumption has been confirmed with the help of nanoindention tests with high hardness values above 10 GPa, which correspond to the literature values of Fe_2_Al_5_ [31].

The achieved results will be used in future works, where coextruded steel–aluminum workpieces with already existing metallurgical bonds will be forged and heat-treated using tailored forming technology [21]. The investigations will focus on the evolution of the intermetallic layer over the considered process. The use of initially bonded workpieces serves as an advantage for the joint, especially in the areas with lower deformation degrees at the bottom part of the investigated bearing bushing, where no metallurgical joint was observed after compound forging. As a result, the potential of tailored forming technology as an approach for achieving high bond quality along the entire joining zone will be examined within the investigated hybrid bearing bushing.

## Figures and Tables

**Table 1:** Intermetallic phases and superstructures of the type Fe_x_Al_y_ [17,18,19].

Phase	Lattice Structure	Al Content, at.%	Hardness, HV	Density, g/cm^3^
α-Fe	Body-centered cubic (BCC)	0–44.6	Up to 140	7.90
Fe_3_Al	Ordered BCC	23.6–34.1	250–350	6.67
FeAl	Ordered BCC	23.3–54.9	400–520	5.37
Fe_2_Al_7_	Complex BCC	63.0	650–680	Not specified
FeAl_2_	Triclinic	65.6–66.9	1000–1050	4.36
Fe_2_Al_5_	Orthorhombic	70.0–73.3	1000–1050	4.11
FeAl_3_	Monoclinic	74.5–76.6	820–1100	3.95
Al	Face-centered cubic (FCC)	99.998–100	Up to 40	2.7

**Table 2:** Flow stresses of steel AISI 4820 and aluminum 6082 at investigated temperatures.

Material	Temperature, °C	Strain Rate, s^−1^	Flow Stress, MPa
AISI 4820	700–750	1	150–220
		10	200–280
6082	450–500	1	~30
		10	~40

**Table 3:** Results of EDS spectrum analysis for the steel–aluminum joint.

Measurement Region	Material	Elemental Distribution, at.%					
		Al	Mg	Si	Fe	Cr	Mn
1	6082	98.80	0.79	0.04	0.37	-	-
2	AISI 4820	-	-	0.66	97.93	0.87	0.54
3	Intermetallic layer	71.06	-	1.48	26.90	0.33	0.23

## Data Availability

The data presented in this study are available on request from thecorresponding author. The data are not publicly available due to privacy.
